# Developing a Novel Parameter Estimation Method for Agent-Based Model in Immune System Simulation under the Framework of History Matching: A Case Study on Influenza A Virus Infection

**DOI:** 10.3390/ijms18122592

**Published:** 2017-12-01

**Authors:** Tingting Li, Zhengguo Cheng, Le Zhang

**Affiliations:** 1 College of Mathematics and Statistics, Southwest University, Chongqing 400715, China; 2 College of Computer and Information Science, Southwest University, Chongqing 400715, China; czg123@email.swu.edu.cn; 3 College of Computer Science, Sichuan University, Chengdu 610065, China

**Keywords:** agent-based models, generalized additive model, history matching, particle swarm optimization algorithm

## Abstract

Since they can provide a natural and flexible description of nonlinear dynamic behavior of complex system, Agent-based models (ABM) have been commonly used for immune system simulation. However, it is crucial for ABM to obtain an appropriate estimation for the key parameters of the model by incorporating experimental data. In this paper, a systematic procedure for immune system simulation by integrating the ABM and regression method under the framework of history matching is developed. A novel parameter estimation method by incorporating the experiment data for the simulator ABM during the procedure is proposed. First, we employ ABM as simulator to simulate the immune system. Then, the dimension-reduced type generalized additive model (GAM) is employed to train a statistical regression model by using the input and output data of ABM and play a role as an emulator during history matching. Next, we reduce the input space of parameters by introducing an implausible measure to discard the implausible input values. At last, the estimation of model parameters is obtained using the particle swarm optimization algorithm (PSO) by fitting the experiment data among the non-implausible input values. The real Influeza A Virus (IAV) data set is employed to demonstrate the performance of our proposed method, and the results show that the proposed method not only has good fitting and predicting accuracy, but it also owns favorable computational efficiency.

## 1. Introduction

Because of the complexity and nonlinearity, precise computational modelling of natural immune systems is virtually impossible. Removing all of the possible details and retaining only the essential interactions provides a possibility to solve this problem. Differential equation (DE) and agent-based modeling (ABM) are two commonly used simulation techniques for immune systems. DE provides mathematical models to describe the dynamic interactions among the components in the immune system, and the immune response can be easily quantified after estimating the control parameters by fitting the experimental data [1,2,3,4,5,6,7]. But, when faced with the simulation of complex phenomena, DE may fall short in constructing a sufficient biological model. When compared to DE, ABM has its benefit from its great flexibility in tuning the complexity of the agents and can be employed to reflect the real sophisticated system [8,9,10,11,12,13,14,15,16,17,18,19]. However, because ABM describes the system at the level of its constituent units, but not at the top level, it is difficult for ABM to estimate the key parameters by incorporating the experimental data.

In view of this, Tong et al. [20] developed an innovative approach, IABMR, by integrating the advantages of both DE and ABM. Firstly, they denoted each cell as an agent with three phenotypes (i.e., quiescence, proliferation, and apoptosis) and employed ABM to describe the dynamic interactions among the components (i.e., epithelial cells, infected epithelial cells, and virus) and simulate the immune system. Then, they employed local regression (LOESS) to build a regression model that is based on the input and output of ABM. Lastly, the model’s key parameters were optimized using the particle swarm optimization algorithm (PSO) [21,22,23,24,25,26] by fitting the experimental data. IABMR can not only has the potential to simulate the immune system, but it can also infer the model parameters, like DE. The case study of influenza A virus (IAV) infection [1,2] demonstrated its reliability and efficiency.

However, when the dimension of the input becomes large, the LOESS may severely suffer from “curse of dimensionality” [27]. The model estimation would incur great variabilities. The dimension-reduced type model, generalized additive model (GAM) [28,29,30], is usually a popular alternative. GAM is a nonparametric regression modelling technique that is not restricted by linear relationships, and it is flexible regarding the statistical distribution of the data. On the other hand, although the PSO algorithm is very efficient and convenient when solving the optimization problem, it could be hard to discover the acceptable region of the input space because the acceptable values may hide in a tiny proportion of the initially specified input space. In addition, it will take much time to find the optimal solution if the input region of each parameter is wide.

In this work, a systematic procedure for immune system simulation by integrating the advantages of ABM and GAM under the framework of history matching [31] is developed to address all of the issues mentioned above. A novel parameter estimation method by incorporating the experimental data for the simulator ABM during the procedure is proposed. IAV infection data [2,20] is employed to evaluate the efficiency and accuracy of the proposed method. First, we employed ABM as simulator [31] to simulate the data. Then, GAM is employed as the emulator [31] to train a statistical regression model of the simulator using the input and output data of ABM. Next, input space is reduced by discarding the implausible input values using an implausibility measure. At last, the model’s key parameters are optimized using PSO by fitting the experimental data among the retained non-implausible input values.

The results demonstrate that our proposed method not only has good fitting accuracy and prediction precision, and thus possesses good potential in both simulating the immune system and fitting the real experimental data, like IABMR, but also needs less computing cost and owns more computational efficiency than IABMR.

## 2. Results and Discussion

**Figure 1:**
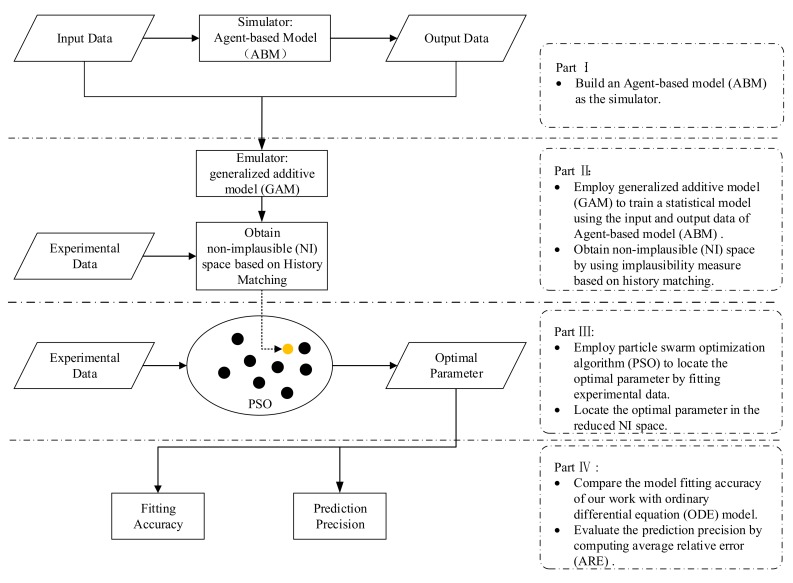
Procedure route.

Figure 1 shows the procedure route of this research under the framework of history matching. The first step is to simulate the human immune system by using a stochastic ABM model as the simulator. Once obtaining the training data from the input and the output data of the simulator ABM, the next step is to convert the simulator into a statistical GAM model with a higher efficiency, known as emulator. Then, an implausibility measure is introduced. The parameter space is reduced by discarding the implausible input values. At last, the model’s key parameters were optimized using PSO in the non-implausible domain by fitting the real experiment data. In this paper, the real Influeza A Virus (IAV) data set [2,20] is employed to demonstrate the performance of our proposed procedure (Figure 1) and the statements of the detailed method are deferred in Section 3.

### 2.1. Observation Data of Influenza A Virus (IAV)

Table 1 lists the real experimental data [2,20] from infection of mice with the H3N2 influenza virus A/X31 strain from 0 to 5 days which is used to fit the model. In this study, we use the sample mean at each point as our observations $z_{j}$ in the process of history matching.

### 2.2. Sampling Data

As stated in Section 3.4, the maximin Latin Hypercube design method [32] is employed to generate sampling data. To make the range of sampled data adapt to our input space $\left(0,2\theta_{0}\right)$, we use Equation (6) to map the original sampled data. Hereinafter, we use bold symbols to denote vectors distinguished from variables. A set of 40 sampling points is listed in Table 2, which is used as inputs $x_{i}$, i=1,2,…,40 into the simulator ABM described in Section 3.1 to generate the training data for the emulator GAM in Section 3.2. As discussed in Section 3.4, in our case of IAV infection study, the inputs x for the simulator ABM is denoted by a four-dimensional vector θ whose components $\theta_{k}$, k=1,2,3,4 represent proliferation rate, infection rate and death rate per hour for epithelial cells, infected epithelial cells and virus separately. In order to accommodate the randomness of the simulator ABM, we execute K=30 times for each point to make the estimation of ${\hat{g}}_{j}\left(x_{i}\right)$ with sufficient accuracy, where ${\hat{g}}_{j}\left(x_{i}\right)$ represents the sample mean of K=30 simulated samples for the jth output at input points $x_{i}$. In our study, 10 outputs are selected from the outputs of the simulator ABM corresponding to the first 10 time points of the real data listed in Table 1.

### 2.3. Non-Implausible Space

**Figure 2:**
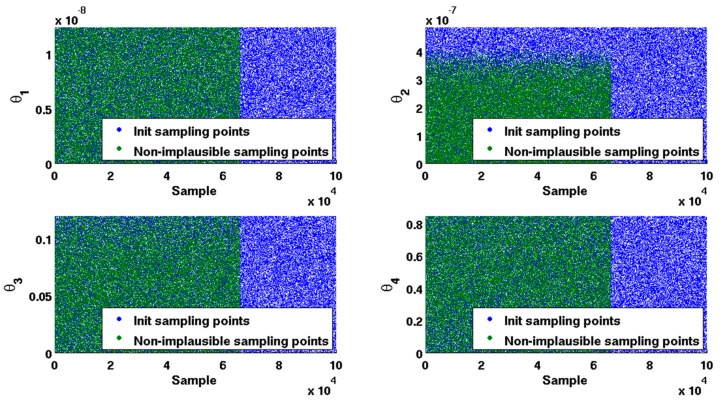
Initial sampling points and non-implausible sampling points.

As discussed in Section 2.2, we can get the dataset $\left(x_{i},{\hat{g}}_{j}\left(x_{i}\right)\right)$, i=1,2,…,40, j=1,2,…,10 from the simulator ABM. Then, the emulator GAM model $M_{0}$ for each output is built based on these simulated data. After that, another set of 10^5^ points, namely $H_{1}$, are drawn from a four-dimensional uniform distribution within the region $\left(0,2\theta_{0}\right)$, and the implausibility measure is evaluated for each point of $H_{1}$. Since we have 10 outputs in our study, for simplicity, we use the following implausibility measure

(1) $$I\left(x\right)=\underset{j=1,2,\ldots ,10}{\max}I_{j}\left(x\right)$$

where $I_{j}\left(x\right)$ is defined by Equation (5) in Section 3.3. According to Pukelsheim’s 3σ rule [33], all $x\in H_{1}$ with I(x)>3 will be deemed implausible. Those non-implausible points that passed the implausibility test will be remained. Comparisons between the initial 10^5^ sampling points and non-implausible sampling points for each parameter are shown in Figure 2.

As shown in Figure 2, when compared to the initial $10^{5}$ sampling points, the non-implausible sampling points shrunk by approximately 34.05%, which is retained to form the non-implausible interval for each parameter. Comparisons between the initial interval and non-implausible interval for each parameter are given in Table 3. It is observed that non-implausible intervals for four parameters have been narrowed.

### 2.4. Fitting Experimental Data

As stated in Section 3.4, once we obtain the reduced non-implausible region for the parameters, the optimization method PSO is employed within the non-implausible region to find the estimated parameters by fitting the experimental data Table 1. Considering that PSO is a stochastic optimization technique, we run the PSO algorithm 50 times and 50 parameter estimates are obtained. The initial parameters and the mean with standard error of the 50 parameter estimates are listed in Table 4.

**Figure 3:**
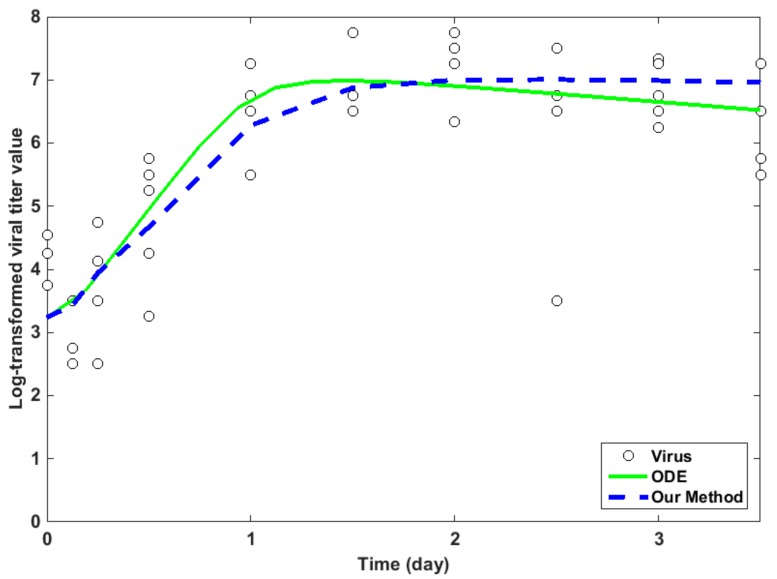
Fitting accuracy of the proposed method and ordinary differential equation (ODE) method [2].

In order to evaluate the fitting efficiency of the proposed model, we draw the fitting at the first 10 timepoints, with the real experimental data in Table 1. The well-developed ordinary differential equation method (ODE) [2] is also employed for comparison. The results are illustrated in Figure 3. It is shown that the two methods have comparable fitting accuracy.

### 2.5. Average Relative Error 

**Figure 4:**
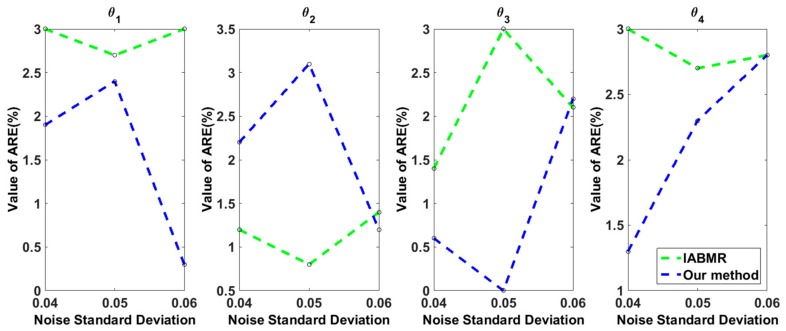
Average relative error (ARE) of IABMR [20] and our proposed method for each parameter with three different level of random noises.

In order to evaluate the prediction accuracy of the whole procedure, we compute the average relative error (ARE) for each parameter $\theta_{k},k=1,2,3,4$, as defined by Equation (8) in Section 3.4, with three different level of random noises 0.04, 0.05, and 0.06. The ARE of the IABMR method [20] is also computed for comparison. The results for each parameter are shown in Figure 4. It is observed that our method have relatively smaller AREs than IABMR, which suggests that our proposed method have favorable prediction precision.

## 3. Methods

### 3.1. Simulator: Using ABM (Agent-based Model) to Simulate the Immune System

A simulator is a computer model to describe the physical process. The simulator output is composed of a vector of r quantities, which is denoted with the vector $f\left(x\right)=[f_{1}\left(x\right),\ldots ,f_{r}\left(x\right)]\in R^{r}$. To accommodate the randomness, we run the simulator K times at each fixed input vector x and the observed values would satisfy.

(2) $$f_{jk}\left(x\right)=g_{j}\left(x\right)+\varepsilon_{jk},\;j=1,2,\ldots ,r,k=1,2,\ldots ,K$$

where $g_{j}\left(x\right)$ is the mean value of the jth output at input value x and $\varepsilon_{jk}$ is a random variable with expectation 0. The training data point for input x is then $\left(x,{\hat{g}}_{j}\left(x\right)\right)$, where ${\hat{g}}_{j}\left(x\right)$ is the sample mean of K simulated outputs $f_{jk}\left(x\right)$ as an estimator of the true mean value $g_{j}\left(x\right)$.

For the case of IAV infection study, Tong et al. [20] developed an ABM to describe the dynamic interactions among the components (i.e., epithelial cells, infected epithelial cells, and virus) by denoting each cell as an agent with three phenotypes (i.e., quiescence, proliferation, and apoptosis). Then, the real experiment data from infection of mice with the H3N2 influenza virus A/X31 strain from 0 to 5 days [2,20] listed in Table 1 is used to evaluate the fitting accuracy. In this work, we simulate the immune system by using the ABM described by [20] as our simulator to obtain the training data for the following emulator.

### 3.2. Emulator: GAM Model

Suppose that y is a vector of observed responses and that $x_{1},x_{2},\cdots ,x_{p}$ are p independent variables. The GAM postulates that the response is additively related to the independent variables via the equation

(3) $$h\left(\mu \right)=\sum_{k=1}^{p}m_{k}\left(x_{k}\right)$$

where $\mu =E\left(y|x_{1},x_{2},\cdots ,x_{p}\right)$ is the expectation of the responses that are conditioned on the predictors. The function h is some known function, called the link function. Each function $m_{k},k=1,2,\ldots ,p$ is assumed to be an unknown nonparametric smooth function, which is needed to be estimated from the observed data. When compared with the LOESS model, even if the number of predictors p is large, the inputs in the functions $m_{k}$ in GAM (2) is still of only one dimension. Therefore, GAM (2) is a dimension-reduced type regression model. Because of the flexibility that is provided by allowing $m_{k}$ to be nonparametric functions, as well as the fact that it is easy to fit these models using standard statistical software, such as S-Plus and R, the GAM has become a powerful analytic tool with a wide range of applications [34,35,36].

In this paper, the simulated data $\left(x,{\hat{g}}_{j}\left(x\right)\right)$ obtained in Section 2.2 is applied to train the GAM emulators for the jth output.

### 3.3. Reducing the Input Space by Using Implausibility Measure

Traditional optimization method is to initialize the parameters’ range first, and then process the optimization. However, it would take a long time to finish the task when the model has several key parameters. Since most of the optimization methods can only find the optima locally, it would probably not give the desirable result when the search ranges of the input parameters are wide.

History matching [31] is designed to identify the set of inputs that would give rise to acceptable matches between the model outputs and the observed data. It provides a tractable calculation involving expectations and variances by excluding implausible parts of the input space that are unlikely to make a good match with the observed data. History matching has several advantages. First, the calculations that are involved in history matching are far more efficient and straightforward to implement. Second, it is possible to exclude implausible space without taking the full sets of inputs and outputs simultaneously into consideration.

As demonstrated in [31], history matching assumes the existence of a physical process that is measured through observations z, which is linked to the best simulator input denoted by $x^{*}$ via

(4) $$z=g\left(x^{*}\right)+\varphi +\varepsilon +\delta$$

where φ and δ are vectors of errors representing Observation Uncertainty (OU) and Model Discrepancy (MD), respectively. OU refers to the uncertainty involved in the data from observations and MD refers to the imperfect representation of reality for simulator [31]. ε is the random error in (1) whose variability would affect the search of implausible values which is referred to Ensemble Variability (EV). These errors OU, MD, and EV are considered to affect the search for the non-implausible input spaces.

For the purpose of evaluating whether the simulator’s output at input value x would result in an acceptable match with the observed data, Andrianakis et al. [31] introduced the implausibility measure given the current uncertainties as follows:

(5) $$I_{j}\left(x\right)=\frac{\left|z_{j}-E^{*}\left[g_{j}\left(x\right)\right]\right|}{{\left[V_{0}+V_{c}\left(x\right)+V_{s}+V_{m}\right]}^{1/2}}$$

Here, $z_{j}$ represents the jth output for the observation data. $E^{*}\left[g_{j}\left(x\right)\right]$ represents the expectation that is obtained by the emulator—GAM model at input point x for the jth output. $V_{0}$ represents the variance that is associated with OU and it equals to the variance of the observation data. $V_{c}(x)$ denotes the code uncertainty as quantified by the emulator—GAM model (2). The variabilities incurred by EV and MD are represented by $V_{s}$ and $V_{m}$, respectively. According to the suggestions provided by [31], $V_{s}$ is set equal to the sample variance of K simulated samples for each input. $V_{m}$ is considered to be equal to 10% of the variance of the simulator output data at all of the design points for simplicity.

A large value of $I_{j}(x)$ would indicate that despite the uncertainties that are present in the system, the prediction about the simulator’s output for x is so far from the observed value $z_{j}$. In this paper, we use 3 as a cut off value according to Pukelsheim’s 3σ rule [33], i.e., all x with $I_{j}(x)>3$ will be deemed implausible. After reducing the implausible points, the input space would be narrowed.

### 3.4. Parameter Estimation

Once we obtain the non-implausible parameter space, the optimization method PSO is employed to locate the estimated parameters by fitting the observation data.

In our case of IAV infection study, the input parameter of ABM is denoted by a four-dimensional vector θ, whose components $\theta_{k}$, k=1,2,3,4 represent proliferation rate, infection rate, and death rate per hour for epithelial cells, infected epithelial cells, and virus separately. The definitions are given in Section 2.2 and Table 1 in [20]. According to [2], θ are estimated as ($6.2\;\times \;10^{-9}$, $2.47\;\times \;10^{-7}$, $5.98\;\times \;10^{-2}$, $4.23\;\times \;10^{-1}$). We refer to this as initial parameter $\theta_{0}$, and set the input parameter space of ABM within the region $\left(0,2\theta_{0}\right)$. In this research, the inputs from the parameter vector space for the simulator ABM are obtained by the maximin Latin Hypercube design method [32], which can generate uniformly distributed points in the input space (0, 1). To adapt to our input space $\left(0,2\theta_{0}\right)$, the sampling inputs that are obtained by the maximin Latin Hypercube design method are mapped to the region $\left(0,2\theta_{0}\right)$ via the mapping function:

(6) H=(b−a)d+a

where d∈(0, 1) and $a=0,b=2\theta_{0}$.

Once the sampling input points are obtained, the simulator runs at the selected inputs to get the training dataset for emulator. Then, GAM model $M_{0}$ is built by using R function **gam()**. After that, we regenerate another sampling data set $H_{1}$ within the region $\left(0,2\theta_{0}\right)$ and take $H_{1}$ as input into $M_{0}$ to obtain prediction output data $G_{1}$. The implausibility measure (4) is employed to evaluate each point of $H_{1}$ and those that do not pass the implausibility test are deemed implausible, meaning that the simulator cannot match the observations given the current error specifications. The initial input space $\left(0,2\theta_{0}\right)$ would be reduced to be a subset of non-implausible space. Next, PSO is employed to locate the optimal parameter $\theta^{*}$ within the non-implausible space by fitting the real experimental data, i.e., the estimated parameter $\theta^{*}$ is obtained by minimizing the following objective function

(7) $$\sum_{j=1}^{m}{\left(z_{j}-{\hat{g}}_{j.GAM}\left(x\right)\right)}^{2}$$

with respect to x where m is the number of outputs and ${\hat{g}}_{j.GAM}\left(x\right)$ is the fitted value from the GAM model $M_{0}$ at input x.

In order to evaluate the prediction accuracy of the whole procedure, we repeat the whole procedure, except for that the normal distributed error is used to add noise for each replicate of the data set obtained by the simulator ABM with key parameter given by $\theta^{*}$.

Finally, we compute the average relative error (ARE) [3] of our procedure via

(8) $$\mathrm{ARE}=\sum_{i=1}^{M}\frac{\left|\theta_{i}^{*}-\theta^{*}\right|}{M\times \left|\theta^{*}\right|}\times 100\%$$

Here, $\theta_{i}^{*}$ denotes the optimal local parameter of each replicate and M represents the number of total replicates. ARE is widely used to evaluate the prediction of statistical models. The smaller the ARE value is, the better the model performs.

## 4. Conclusions

In this work, we proposed a systematic procedure for immune system simulation by integrating ABM and GAM under the framework of history matching. Because of its great flexibility, ABM has been widely used to simulate the biological immune system. However, it is crucial for ABM to obtain an appropriate key parameter by incorporating the real experimental data. One previous study [20] proposed an innovative method IABMR by employing the LOESS model and PSO optimization method. In this research, the dimension-reduced type regression model GAM (2) is employed to avoid “curse of dimensionality” in LOESS to help increase the precision of the model. Moreover, to further improve the computation efficiency during the optimization, the search region for the parameters is reduced by discarding those inputs that have large implausibility measure and would give rise to unacceptable matches between the model outputs and the observed data.

In our case of IAV infection data (Table 1), we historically match a four inputs and 10 outputs simulator by integrating ABM and GAM. By computing the implausibility measure for each point that was sampled from the initial parameter space, the non-implausible input space shrank by 34.05% (Figure 2). In our computation, during processing the optimization PSO by fitting the real data, IABMR [20] needs 3465 runs by sampling 385 inputs and running nine times at each input to make the PSO converge, but our method just needs 1200 runs, which shows that our method has more computation efficiency than IABMR when locating key parameters. Meanwhile, with regard to fitting accuracy, our proposed method is comparable to the previous well-developed ODE model [2] (Figure 3). The average relative error (ARE) is widely used to evaluate the prediction of statistical models. The smaller the ARE value is, the better the model performs. When compared with IABMR [20], our method also shows favorable prediction accuracy (Figure 4).

However, [37] suggested that in the process of history matching during our procedure for immune system simulation, several computation waves might be needed to further reduce the parameter space in order to locate more precise parameters during optimization. Nevertheless, the simulator ABM at each wave during history matching does cost so much computing resource. It may be of use to bring in parallel computation, such as graphics processing unit technology [18,37,38] to accelerate the procedure. For example, each input of ABM needs to execute K times at each wave during history matching. If we could map the whole computing job to several nodes, it would save much time. The detailed work on how to make the simulator ABM during history matching in our procedure of immune system simulation run at parallel nodes still needs much to rewrite codes and it is out of the scope of this research. We leave this as our future work.

In conclusion, in this study a systematic procedure for immune system simulation by integrating the advantages of ABM, GAM under the framework of history matching is proposed. The estimation for the key parameters to incorporate the real experiment data for the simulator ABM in the procedure is developed. The real data analysis demonstrates that our study has good efficiency and accuracy, and thus could mimic the immune system on multiple levels.

## Figures and Tables

**Table 1:** Real experimental data from 0 to 5 days.

	Time Points (Day^−1^)												
Samples	0	0.125	0.25	0.5	1	1.5	2	2.5	3	3.5	4	4.5	5
1	4.25	2.5	3.5	4.25	5.5	6.5	6.33	6.75	6.5	6.5	6.5	7	6.33
2	3.75	2.5	4.75	3.25	6.75	6.75	7.5	3.5	7.33	7.25	6.25	6.5	5.5
3	4.25	3.5	4.75	5.25	6.5	7.75	7.75	7.5	7.33	7.25	6.5	6.25	5.75
4	3.75	3.5	4.13	5.75	7.25	NA	7.25	6.5	6.25	5.5	NA	NA	NA
5	4.55	2.75	2.5	5.75	NA	NA	NA	7.5	6.75	6.5	NA	NA	NA
6	4.25	NA	4.75	5.5	NA	NA	NA	NA	7.25	5.75	NA	NA	NA

**Table 2:** Sampling data set as inputs into the simulator ABM, where $\theta_{k}$, k=1,2,3,4 represent proliferation rate, infection rate and death rate per hour for epithelial cells, infected epithelial cells, and virus, separately.

Samples	$\theta_{1}$	$\theta_{2}$	$\theta_{3}$	$\theta_{4}$
1	3.466758 × 10^−9^	2.288938 × 10^−7^	2.460326 × 10^−2^	8.616152 × 10^−2^
2	8.001264 × 10^−9^	4.300130 × 10^−7^	8.741329 × 10^−2^	3.955367 × 10^−1^
3	1.081166 × 10^−8^	1.932323 × 10^−7^	1.004010 × 10^−1^	3.100995 × 10^−1^
4	1.090549 × 10^−8^	2.812863 × 10^−7^	8.654013 × 10^−2^	3.202220 × 10^−1^
5	9.102252 × 10^−9^	4.513295 × 10^−7^	4.608862 × 10^−2^	1.196989 × 10^−1^
6	3.405003 × 10^−9^	3.370440 × 10^−8^	1.130993 × 10^−1^	6.104288 × 10^−1^
7	8.092254 × 10^−9^	4.017315 × 10^−8^	2.174145 × 10^−2^	2.430601 × 10^−1^
8	2.010234 × 10^−9^	1.745676 × 10^−8^	6.418247 × 10^−2^	1.722317 × 10^−1^
9	1.691198 × 10^−9^	3.527068 × 10^−7^	1.158533 × 10^−1^	1.302177 × 10^−2^
10	2.912003 × 10^−9^	2.957414 × 10^−7^	2.715800 × 10^−2^	2.602361 × 10^−1^
11	2.554265 × 10^−9^	9.798854 × 10^−8^	1.866300 × 10^−2^	5.577698 × 10^−1^
12	6.864842 × 10^−9^	4.184238 × 10^−7^	1.079778 × 10^−1^	7.730243 × 10^−1^
13	1.121311 × 10^−9^	3.102666 × 10^−7^	2.784293 × 10^−3^	5.343298 × 10^−2^
14	9.583759 × 10^−9^	6.668325 × 10^−8^	3.832125 × 10^−2^	7.836137 × 10^−1^
15	8.762499 × 10^−9^	5.740286 × 10^−8^	6.656615 × 10^−2^	1.462840 × 10^−1^
16	1.167708 × 10^−8^	1.339571 × 10^−7^	1.286682 × 10^−2^	7.554816 × 10^−1^
17	5.319678 × 10^−9^	4.057522 × 10^−7^	7.242679 × 10^−2^	6.884958 × 10^−1^
18	7.634766 × 10^−9^	8.162650 × 10^−8^	9.417931 × 10^−2^	8.124229 × 10^−1^
19	9.973253 × 10^−9^	1.641823 × 10^−7^	5.553776 × 10^−2^	1.506380 × 10^−1^
20	6.455279 × 10^−9^	1.729994 × 10^−7^	7.591240 × 10^−2^	4.765285 × 10^−1^
21	3.989849 × 10^−9^	9.193181 × 10^−8^	9.013124 × 10^−2^	4.137486 × 10^−1^
22	1.212724 × 10^−8^	4.454997 × 10^−7^	1.593432 × 10^−2^	1.957182 × 10^−1^
23	9.163350 × 10^−10^	3.647394 × 10^−7^	7.019446 × 10^−2^	5.823037 × 10^−1^
24	4.437117 × 10^−9^	3.801312 × 10^−7^	8.076542 × 10^−3^	6.162935 × 10^−1^
25	1.186253 × 10^−8^	3.221797 × 10^−7^	6.068513 × 10^−2^	2.227833 × 10^−1^
26	5.134477 × 10^−9^	2.635175 × 10^−7^	9.752898 × 10^−2^	5.182151 × 10^−1^
27	4.222250 × 10^−9^	2.151502 × 10^−7^	1.059426 × 10^−1^	8.356908 × 10^−1^
28	1.246306 × 10^−9^	3.445810 × 10^−7^	5.116530 × 10^−2^	4.604467 × 10^−1^
29	1.134631 × 10^−8^	1.157556 × 10^−7^	3.036958 × 10^−2^	6.538406 × 10^−1^
30	2.343542 × 10^−9^	1.483302 × 10^−7^	7.804259 × 10^−2^	4.427248 × 10^−1^
31	4.911390 × 10^−9^	1.124756 × 10^−8^	1.018424 × 10^−1^	2.351053 × 10^−2^
32	1.043893 × 10^−8^	4.616473 × 10^−7^	5.736185 × 10^−2^	2.911685 × 10^−1^
33	5.792641 × 10^−9^	4.757734 × 10^−7^	1.195959 × 10^−1^	7.008772 × 10^−1^
34	7.162705 × 10^−9^	1.314465 × 10^−7^	1.195076 × 10^−2^	3.444968 × 10^−1^
35	6.743423 × 10^−9^	2.437133 × 10^−7^	5.718468 × 10^−3^	6.614001 × 10^−2^
36	8.583690 × 10^−9^	3.363820 × 10^−7^	3.510043 × 10^−2^	4.877613 × 10^−1^
37	1.832510 × 10^−10^	1.983512 × 10^−7^	4.478183 × 10^−2^	3.777456 × 10^−1^
38	5.980517 × 10^−9^	3.927292 × 10^−7^	4.956199 × 10^−2^	6.684690 × 10^−1^
39	5.754720 × 10^−10^	2.763359 × 10^−7^	4.084022 × 10^−2^	5.291611 × 10^−1^
40	9.649890 × 10^−9^	2.419316 × 10^−7^	8.210135 × 10^−2^	7.266126 × 10^−1^

**Table 3:** Initial interval and non-implausible interval for each parameter.

Parameters	Initial Interval	Non-Implausible Interval
$\theta_{1}$	[0, 1.240000 × 10−8]	[3.8139 × 10−14, 1.2400 × 10−8]
$\theta_{2}$	[0, 4.840000 × 10−7]	[2.5844 × 10−14, 4.8400 × 10−7]
$\theta_{3}$	[0, 1.196000 × 10−1]	[8.7906 × 10−7, 1.1960 × 10−1]
$\theta_{4}$	[0, 8.460000 × 10−1]	[6.1473 × 10−6, 8.4600 × 10−1]

**Table 4:** Initial parameters and the means with standard errors in brackets of the 50 parameter estimates.

Parameters				
Model	$\theta_{1}$	$\theta_{2}$	$\theta_{3}$	$\theta_{4}$
Initial Parameters	6.2000 × 10^−9^	2.4200 × 10^−7^	5.9800 × 10^−2^	4.2300 × 10^−1^
Our Estimates	6.5656 × 10^−9^	7.2467 × 10^−9^	2.7739 × 10^−2^	1.2595 × 10^−1^
	(4.2290 × 10^−9^)	(6.4759 × 10^−11^)	(2.8178 × 10^−7^)	(3.1538 × 10^−6^)
